# Drug repurposing for COVID-19: a potential threat of self-medication and controlling measures

**DOI:** 10.1136/postgradmedj-2020-138447

**Published:** 2020-08-26

**Authors:** Tauqeer Hussain Mallhi, Yusra Habib Khan, Nasser Hadal Alotaibi, Abdulaziz Ibrahim Alzarea, Abdullah Salah Alanazi, Sumera Qasim, Muhammad Shahid Iqbal, Nida Tanveer

**Affiliations:** Department of Clinical Pharmacy, College of Pharmacy, Jouf University, Sakaka, Al-Jouf, Kingdom of Saudi Arabia; Department of Clinical Pharmacy, College of Pharmacy, Jouf University, Sakaka, Al-Jouf, Kingdom of Saudi Arabia; Department of Clinical Pharmacy, College of Pharmacy, Jouf University, Sakaka, Al-Jouf, Kingdom of Saudi Arabia; Department of Clinical Pharmacy, College of Pharmacy, Jouf University, Sakaka, Al-Jouf, Kingdom of Saudi Arabia; Department of Clinical Pharmacy, College of Pharmacy, Jouf University, Sakaka, Al-Jouf, Kingdom of Saudi Arabia; Department of Pharmacology, College of Pharmacy, Jouf University, Sakaka, Al-Jouf, Kingdom of Saudi Arabia; Department of Clinical Pharmacy, College of Pharmacy, Prince Sattam Bin Abdulaziz University, Al Kharj, Kingdom of Saudi Arabia; Women Medical Officer, Tehsil Headquarter Hospital, Jaranwala, Faisalabad, Punjab, Pakistan

Dear Editor,

We read with great interest a recent editorial published in *Postgraduate Medical Journal*,^1^ suggesting the potential harms associated with the use of unproven antimicrobials for the management of COVID-19. We appreciate the efforts of authors to underscore the potential drug safety concerns during the ongoing health crisis. In align with these suggestions, we felt inclined to share another potential hazard associated during the current phase of drug repurposing.

COVID-19 has encompassed more than 200 countries with unprecedented rise in infected cases along with mortalities. Healthcare professionals and researchers around the globe are battling hard to combat the severe acute respiratory syndrome coronavirus 2 (SARS-CoV-2) and taking all possible measures to reduce the growing encumbrance of the disease. Since most of the countries have imposed lockdown and people are confining to their homes with restricted movements, they are more vigilant for the updates on COVID-19, particularly on treatments and preventive measures. Most of the people are relying on electronic media and social networking to get latest and updated information related to the disease. On the other hand, increasing numbers of COVID-19-related deaths are creating panic among the general public. In such circumstances, both general and healthcare communities are anxiously waiting to receive any positive news regarding the treatment and prevention of infection. Literature has reported several medications such as hydroxychloroquine (HCQ) and chloroquine (CQ)^2^ carrying the potential to cure COVID-19. Most of these reports primarily rely on invitro investigations and lack firm clinical evidences from human studies.^3^  ^4^  ^5^ Though such a hypothesis, generations for the treatment of COVID-19 are the need of hour, but uncontrolled amplified dissemination of news or information by the lay press, electronic and social media may trigger self-medication of candidate drugs among general community. It is pertinent to mention that every news channel or agency is efficiently engaged to break any new findings or studies related to the treatment and prevention of COVID-19. Such news reports may influence the people who are searching for appropriate measures to save themselves from the virus.

## Drug repurposing and potential of self-medication

The discovery of new candidates for the treatment of COVID-19 is improving the clinical management of infected cases but at the same time portending potential risks of self-medication among the general public. Despite the absence of firm evidences, attempts to self-medicate with the repurposed drugs have been observed in the public. HCQ can have life-threatening side effects if the dose is not carefully controlled, and cases of CQ poisoning have been reported in Nigeria and the USA. Nigerian health authorities have already issued several warnings amid CQ poisoning, which immediately occurred after the media reports.^6^ It must be noted that Nigeria is endemic for Malaria, and shortage of CQ or HCQ may result in another parasitic catastrophe. Moreover, sudden interest in HCQ has led to reports of shortages for patients who rely on the drug to treat their autoimmune diseases that could place these patients at risk for severe and even life-threatening flares; some may require hospitalisation when hospitals are already at capacity.^6 7^

Both CQ and HCQ have been approved by the US Food and Drug Administration (FDA) under emergency use authorization and subsequently advocated by the Indian Council for Medical Research. Indian health ministry has also endorsed the prophylactic use of these drugs among healthcare professionals.^8^ During the current environment of global panic, such endorsements may result in widespread use of drugs for prophylaxis and overly optimistic perception related to the effectiveness of CQ and HCQ among the public. Widespread use of HCQ may expose some patients to rare but potentially fatal harms, including serious cutaneous adverse reactions, fulminant hepatic failure and ventricular arrhythmias (especially when used with azithromycin). Furthermore, overdose of these drugs is hazardous and difficult to treat.^3^ Recent recommendations of FDA limit the use of these drugs in hospital setting to treat COVID-19 in adults and adolescents who weigh at least 50 kg and are not able to participate in clinical trials. Moreover, FDA also insisted to obtain these drugs from the national stockpile to protect the supply for other patients who have relied on the drugs for years to control autoimmune diseases including lupus and rheumatoid arthritis. Despite these recommendations, an immediate surge in demand was observed resulting in shortage of both drugs.^9^ The situation of drug shortage in India is not different, probably indicating widespread self-medication. Similar problem is observed in Pakistan, where most of the pharmacies in major cities ran out of these medications immediately after the announcement of their effectiveness in COVID-19. Although both drugs are inexpensive and abundantly available in the market, several companies have also hiked their price.^6^

Ivermectin is another easily available and accessible drug, which has been recently repurposed for the immediate use in treatment in SARS-CoV-2.^4^ This antiparasitic drug also carries substantial odds to be self-medicated attributing to its low price and easy accessibility. Although ivermectin has an established safety profile for human use and is FDA-approved for several parasitic infections,^2^ this drug poses risks of serious neurotoxicity, which can be even fatal in some cases. Moreover, there is no enough evidence to claim this drug is safe for pregnant women.^10^ Azithromycin is another repurposed drug, which is easily accessible at community level and can be self-medicated alone or in combination with HCQ.

## Potential implications mitigating the self-medication risks

The association of serious risks of widespread self-medication and media announcements can be evident by the public interest in CQ and HCQ as demonstrated by the data of Global Google Trends where search for these drugs spiked following the press conference acknowledging the benefits of these drugs for COVID-19.^11^ These patterns underscore that any news regarding the effectiveness of a particular drug for the management of COVID-19 may pose substantial risks of self-medication, which may either result in adverse outcomes, deaths or drug shortage. Even 0.1% drug-associated complications would be thousands in number, which will be difficult to cope by healthcare system currently engaged to combat the COVID-19. Moreover, increased demand for these drugs by the general public may halt the supply and availability of these agents for their approved indications and may also relate to price gouging. These factors may collapse the existing fragile healthcare system in most of the developing or underdeveloped countries during the ongoing pandemic. We believe that careful or responsible reporting and addressing of these potential factors by the health authorities is of paramount importance. We felt inclined to share few suggestions in this regard which should be considered in haste to facilitate the already overwhelmed healthcare system.

- Since repurposing of most of the drugs for COVID-19 is based on either in vitro investigations or findings from small uncontrolled case series, both electronic and paper media should avoid any claim of cure for the general public.
- Media should avoid any exaggerated or amplified statements which could act as triggers for self-medication among general community.
- During the current pandemic, numerous TV channels are hosting talks or live shows on the disease by inviting various stakeholders. These shows or programmes must avoid any discussion on drug use or on the benefits of under-investigated drugs of COVID-19.
- The most reasonable and ethical approach would be limiting the COVID19-related discussions in TV shows to healthcare professionals rather than political or business figures.
- Media regulatory authorities must issue guidelines to host or break any statement on the prevention and treatment of COVID-19.
- Community pharmacists can play a crucial role in current circumstances and may limit the sale of under-investigation drugs by prescription only.
- Pharmacies should control bulk selling of repurposed drugs, even to the patients who are already relying on these drugs for other indications.
- Pharmacists may counsel general community regarding the harms of self-medication and encourage customers to adopt quarantine and control measures for the prevention of disease.
- Pharmacists should vigilantly observe and report any unusual activities in the supply chain, doubted sale pattern in the market and unethical promotion of these drugs.
- The role of drug regulatory authorities is of utmost importance to control the self-medication during current pandemic. Drug regulators can ensure appropriate supply of these drugs particularly in areas of high demand such as hospitals receiving patients with COVID-19 and centers treating malaria or autoimmune diseases. They can strictly observe unusual selling trends of these drugs and may take necessary actions wherever required.

Self-medication during the current pandemic may aggravate the ongoing health crises for which none the country is readily prepared. We believe that restricted and careful media announcements, active involvements of pharmacists and drug regulators with positive support from the national health authorities will mitigate the potential risks of self-medication and subsequent drug shortage and price hike during the current pandemic.
